# Bacterial chondronecrosis with osteomyelitis lameness in broiler chickens and its implications for welfare, meat safety, and quality: a review

**DOI:** 10.3389/fphys.2024.1452318

**Published:** 2024-08-29

**Authors:** Amanda Anthney, Anh Dang Trieu Do, Adnan A. K. Alrubaye

**Affiliations:** ^1^ Center of Excellence for Poultry Science, University of Arkansas, Fayetteville, AR, United States; ^2^ Cell and Molecular Biology Program, University of Arkansas, Fayetteville, AR, United States

**Keywords:** poultry, bacterial chondronecrosis with osteomyelitis, lameness, animal welfare, food safety, meat quality

## Abstract

The exponential increase in global population continues to present an ongoing challenge for livestock producers worldwide to consistently provide a safe, high-quality, and affordable source of protein for consumers. In the last 50 years, the poultry industry has spearheaded this effort thanks to focused genetic and genomic selection for feed-efficient, high-yielding broilers. However, such intense selection for productive traits, along with conventional industry farming practices, has also presented the industry with a myriad of serious issues that negatively impacted animal health, welfare, and productivity–such as woody breast and virulent diseases commonly associated with poultry farming. Bacterial chondronecrosis with osteomyelitis (BCO) lameness is one such issue, having rapidly become a key issue affecting the poultry industry with serious impacts on broiler welfare, meat quality, production, food safety, and economic losses since its discovery in 1972. This review focuses on hallmark clinical symptoms, diagnosis, etiology, and impact of BCO lameness on key issues facing the poultry industry.

## 1 Introduction

Since the 1940s, the poultry industry has consistently led the field of livestock production in terms of volume of meat produced and affordability for consumers annually, the demand of which is projected to continuously grow in upcoming years (USDA, 2023). At a glance, approximately more than nine billion broilers were grown in 2018, to a total of 25.6 billion kilograms in live weight produced with the United States, Brazil, and China leading global production volumes (National Chicken Council, 2021a). Per capita consumption of chicken meat in the United States was estimated at 42.4 kg in the same year and has consistently increased per annum, with 2024 estimation at 45.5 kg (National Chicken Council, 2021b). This popularity has resulted in the making of the modern broiler, which would grow significantly larger in a shorter time frame while improving feed efficiency over the years, thanks to intense genetic selection for productive parameters and advancements in broiler nutrition (Tallentire et al., 2016; Castro et al., 2023). Such a drastic change in broiler strain physiology in the 5 decades from 1957 to 2005 was highlighted by Zuidhof et al. (2014), who reported a 400% increase in broiler growth rate while the FCR is reduced by half. The high production volumes and low consumer prices enjoyed by the poultry industry compared to other major livestock species like beef and pork (USDA, 2024) is largely possible thanks to this outstanding growth rate and extremely efficient feed conversion of the modern broiler–along with a vertically integrated business model employed by the industry (Vukina, 2001) – even with feed ingredient procurement and production often constituting a significant production cost in livestock farming. Unfortunately, this rapid increase in growth rate to meet production and consumption demands, coupled with conventional industrial poultry farming practices, has resulted in significant physiological pressure on the animals, leading to negative impacts on broiler welfare, health, and productivity. For example, the increased prevalence of woody breast syndrome–signified by the drastic hardening of breast muscle tissue–has been linked to multiple causes stemming from physiological, genetic, and production factors (Hoving-Bolink et al., 2000; Sihvo et al., 2018; Tixier-Boichard, 2020; Welter et al., 2022). Similarly, modern poultry farming has also been the subject of some scrutiny, with high stocking densities and poor housing conditions more likely to give rise to rapid pathogenic spread of diseases between flocks and other welfare issues, such as pododermatitis and hock burns (Berg, 2004; Buijs et al., 2009; Jacob et al., 2016).

In the same vein, lameness is one of the most prevalent issues currently affecting modern broiler health and welfare (Julian, 1998; EFSA, 2012; Granquist et al., 2019). More specifically, in addition to common hock burn or pododermatitis-associated lameness that may result in painful gait for the animal, bacterial chondronecrosis with osteomyelitis (BCO) lameness has rapidly become a condition of great concern to the poultry industry due to its increasingly apparent impact on welfare and economic losses since its first reported case in 1972 (Nairn and Watson, 1972), which has been reported in both current literature (McNamee et al., 1998; Cook, 2000; Bradshaw et al., 2002; Dinev, 2009) as well as industrial communications over time. Initiated by leg bone microfractures that worsen as the broiler ages and eventually become bacterial infection sites, BCO lameness results in severely reduced animal mobility, morbidity, and mortality–often with no recourse. Annually, BCO lameness was estimated to result in losses of roughly $100 million to broiler culls in the United States (Cook, 2000; McNamee et al., 2000) and is reported as a common cause of lameness with increasing prevalence in various regions globally (Pattison, 1992; Dinev, 2009; Herdt et al., 2009; Stalker et al., 2010a; Braga et al., 2016; Ekesi et al., 2021). This review will thus focus on several aspects regarding BCO-associated lameness’s potential pathogenesis, clinical symptoms, diagnosis, and implications in relation to economic impacts, food safety, and meat quality concerns.

## 2 Pathogenesis and pathophysiology

### 2.1 Rapid body weight gain and mechanical stress

Current proposed mechanisms of BCO lameness pathophysiology often involve a combination of the rapid weight gain in the modern broiler in a short period of time and the comparatively disproportionate growth rate of the broiler’s skeletal integrity to support it (Wideman and Prisby, 2013; Wideman, 2016). While the average modern broiler will have gained roughly a hundred-fold in body weight by the eighth week of age (Bellairs and Osmond, 2005; Zuidhof et al., 2014; Aviagen Inc, 2022; Cobb-Vantress Inc, 2022), both the femora and tibiae will have only increased by approximately four times in length and about three to five times in mid-shaft diameter in the first 6 weeks by comparison (Applegate and Lilburn, 2002; Damaziak et al., 2019). While exhaustive academic and industrial improvements in genetic selection (Kapell et al., 2012; Siegel et al., 2019) as well as research of broiler nutrition and environmental enrichment (Nakhon et al., 2019; Pedersen et al., 2020; Biesek et al., 2022) have provided valuable insights into healthier broiler leg development, the negative relationship between fast-growing genetics and healthy broiler skeletal and leg bone development remains to this day, where rapid muscling and weight gain are at odds with the latter–particularly during the earlier stage of rapid weight gain (Rath et al., 2000; González-Cerón et al., 2015; Kierończyk et al., 2017; Santos et al., 2022). In the short productive lifetime of the commercial broiler, the long bones of the leg are exposed to excessive mechanical stress from its drastically disproportionate body weight as the broiler locomotes, causing shear stress and torque on the immature skeletal system (Wideman, 2016). Long chondrocyte columns in the proximal growth plates commonly seen in the avian femur and tibia are also especially susceptible to such mechanical stress, causing microfractures between layers of cartilage that often transect blood vessels, causing reduced blood flow and eventual necrosis (McNamee et al., 1998; Wideman and Prisby, 2013; Wideman, 2016; Haynes, 2023). Due to the significantly longer columns of chondrocytes and thicker growth plates in the proximal ends of the femur and tibia, they are far more susceptible to lesions compared to the distal ends. Resultant infection of these osteochondrotic clefts in femoral and tibial proximal growth plates usually follow, whether through bacterial translocation of the broiler’s gastrointestinal and/or respiratory systems, or acquired through the surrounding environment, giving rise to further necrosis–and eventually, the associated lameness (Wideman, 2016). While BCO infections most commonly manifest in the proximal ends of the femur and tibia, they can also be found affecting the fourth thoracic vertebra (T4), causing the condition known as spondylitis, which may be clinically misclassified as “kinky back” – or non-bacterial spondylolisthesis (Carnaghan, 1966; Wideman, 2016; Choppa and Kim, 2023).

### 2.2 Bacterial hematogenous translocation

**FIGURE 1:**
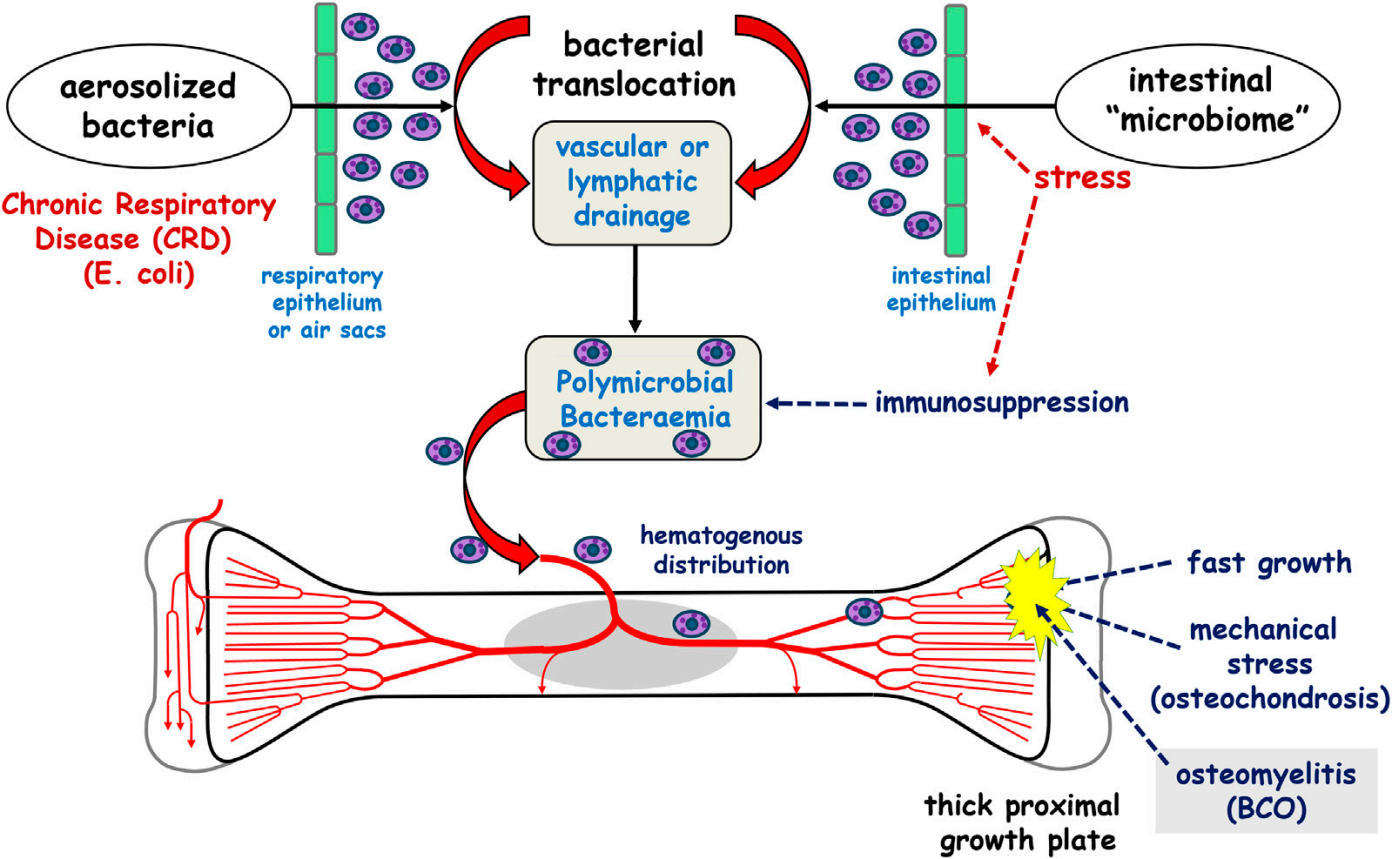
The current postulated hypothesis of bacterial chondronecrosis with osteomyelitis (BCO) pathogenesis. Bacteria translocate from acquired aerosolized bacteria in the respiratory system or from the intestinal microbiome and colonize osteochondrotic clefts in the proximal growth plates on the weight bearing bones of the leg. Reproduced from Wideman (2016) with permission from Elsevier.

The introduction of bacteria to damaged femoral and tibial growth plates, which leads to subsequent worsening infection and necrosis, is linked to the hematogenous translocation of opportunistic bacteria from the intestinal microbiota or aerosolized bacteria from the respiratory system (Wideman, 2016). Figure 1, summarizes the hypothesized hematogenous translocation model.

Overwhelmingly, *Staphylococcus* spp. make up the majority of represented species within BCO lesions, although others like *Enterococcus* spp. and *Escherichia coli* have also been identified (Jiang et al., 2015; Wideman, 2016; Szafraniec et al., 2022). Within the *Staphylococcus* genus, while *S. aureus* has been widely associated with lesions and associated lameness, *S. agnetis* has also been identified as another potential key species of interest due to its unique virulence factor, which is composed of homologs for both pathogenic and non-pathogenic Staphyolococci such as ORF531, ORF1179-1182, and ORF2344, which are also responsible for *Staphylococcus aureus* virulence factors (Al-Rubaye et al., 2015). A similar study at the University of Arkansas successfully demonstrated BCO lameness induction capability of the hypervirulent *Staphylococcus agnetis* strain 908 in drinking water challenges, where tested probiotic preventive measures proved less effective in clinical BCO lameness reduction compared to other *Staphylococcus* species (Al-Rubaye et al., 2017). It has been hypothesized that such evolved virulence may have been the result of intense selection from past focused lameness research efforts using the unique and effective wire-flooring model at the facility (Wideman Jr et al., 2012; Wideman Jr et al., 2013; Al-Rubaye et al., 2015; Al-Rubaye et al., 2017; Shwani et al., 2020).

### 2.3 Gastrointestinal bacterial translocation (“leaky gut”) and respiratory bacterial translocation

One of the prevailing proposed mechanisms behind BCO pathogenesis is the translocation of bacteria from the gastrointestinal tract due to poor integrity of the intestinal barrier, also commonly known as “leaky gut” (Wideman and Prisby, 2013; Wideman, 2016; Ducatelle et al., 2023). The lining of the small intestine comprises a layer of epithelial cells maintained by “tight junctions” (TJ) – or multi-complexes of proteins (such as occludin and claudin) responsible for integrity and permeability of this layer (Tomita et al., 2004). These junctions, along with the associated epithelial layer, are generally considered the first barrier between the small intestine and the bloodstream and is thus subject to disruption from enteric pathogens and other initiating factors (Tomita et al., 2004; Ducatelle et al., 2023). When a broiler is subjected to environmental stress, compromised immunity, or a poor diet, these tight junctions may have reduced functionality, leading to increased permeability between epithelial layers and hematogenous translocation of pathogenic bacteria (Wideman and Prisby, 2013; Wideman, 2016; Alharbi et al., 2024b). This passage from the gut microbiota into the bloodstream can induce inflammation of the gut and surrounding tissues, as well as resultant BCO infection and lameness when bacteria colonize osteochondrotic clefts in the growth plates of weight-bearing bones (Wideman, 2016; De Meyer et al., 2019). It has been reported that supplementation of high-quality essential trace minerals such as copper, manganese, and magnesium in the broiler diet may have a positive effect in the strengthening and increased expression of TJ proteins, leading to decreased chances of translocation events and subsequent clinical BCO lameness incidence (Alrubaye et al., 2020).

Broilers are also often exposed to airborne environmental pathogens in a multitude of environments, including the hatchery or the housing itself, which may potentially result in persistent complications throughout the broiler’s productive life and decreased food safety for the consumers (Quarles and Caveny, 1979; Cox et al., 2002; Heyndrickx et al., 2002). In stressed and immunocompromised birds, aspirated bacteria may colonize the upper respiratory tract’s mucosal surfaces or avian air sacs, and subsequently proliferate. This colonization of opportunistic bacteria allows for translocation into the bloodstream in a similar fashion as the gastrointestinal route, which can lead to BCO infections (Jiang et al., 2015). While current literature regarding BCO-associated lameness remains somewhat scarce in this topic, examination of the avian and poultry respiratory environment in various conditions had been previously conducted using bronchoalveolar lavage fluid (Bottje et al., 1998; Zhou et al., 2023), which may open up future opportunities in this research aspect.

## 3 Symptoms and diagnosis

**FIGURE 2:**
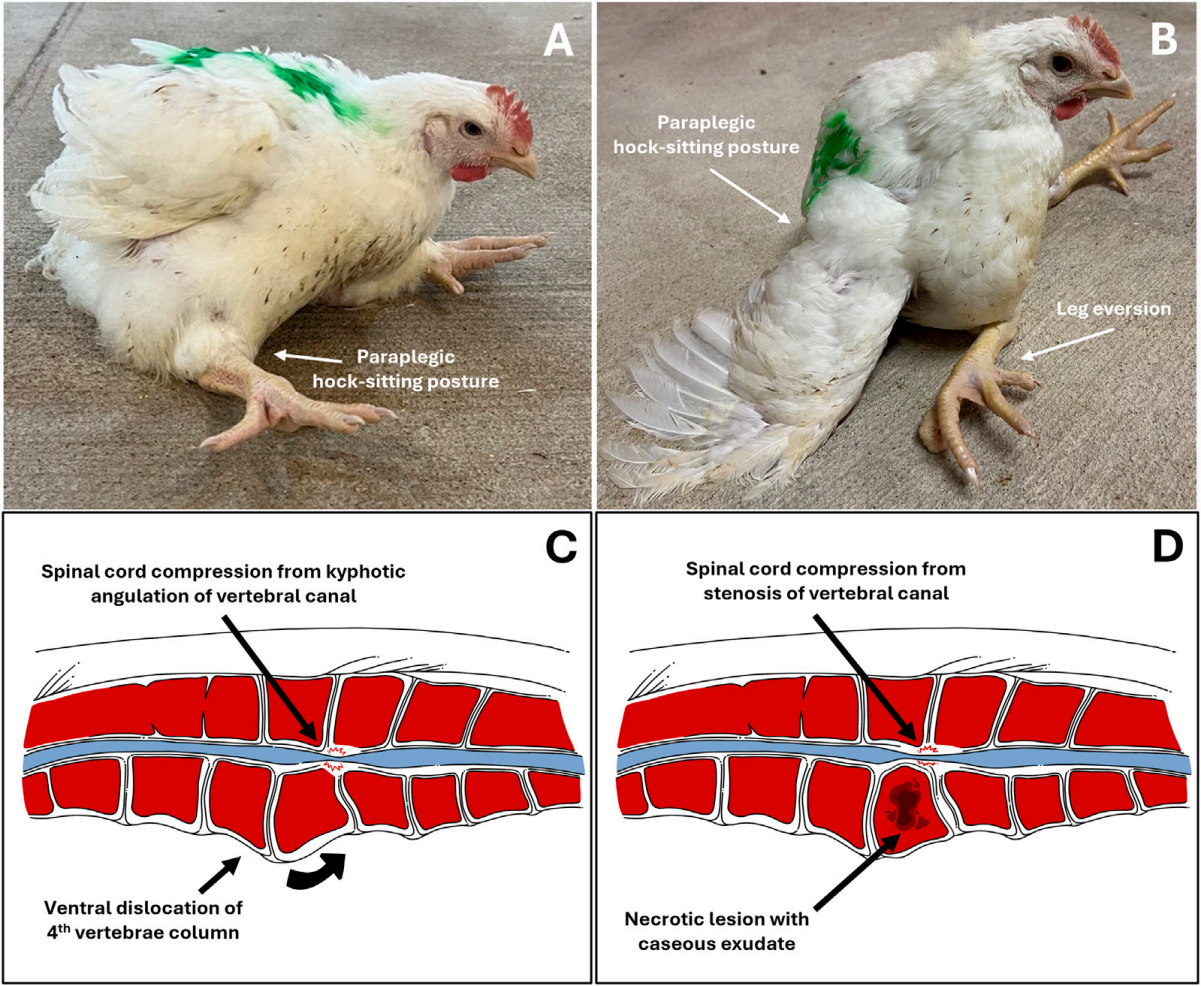
Left to Right, Top Down; **(A,B)** Depictions of broilers exhibiting clinical symptoms of vertebral lameness (spondylitis/spondylolisthesis, “kinky back”) in a paraplegic, hock-sitting posture; **(C,D)** Illustrated anatomical comparison of spondylolisthesis and spondylitis. **(A)**. Typical sitting posture of broilers afflicted with vertebral lameness; **(B)**. Severe vertebral lameness sitting posture, with eversion of the legs; **(C)**. Illustrated transversal cross-section of vertebral column afflicted with spondylolisthesis of the fourth vertebra and compression of the spinal cord in the area due to physical kyphotic angulation of the vertebral canal. Note the relatively intact bone tissue; **(D)**. Illustrated transversal cross-section of vertebral column afflicted with bacterial spondylitis of the fourth vertebral and compression of the spinal cord in the area due to stenosis of the vertebral canal. Note the damaged necrotic bone tissue with caseous exudate within the vertebral body compared to **(C)**.

**FIGURE 3:**
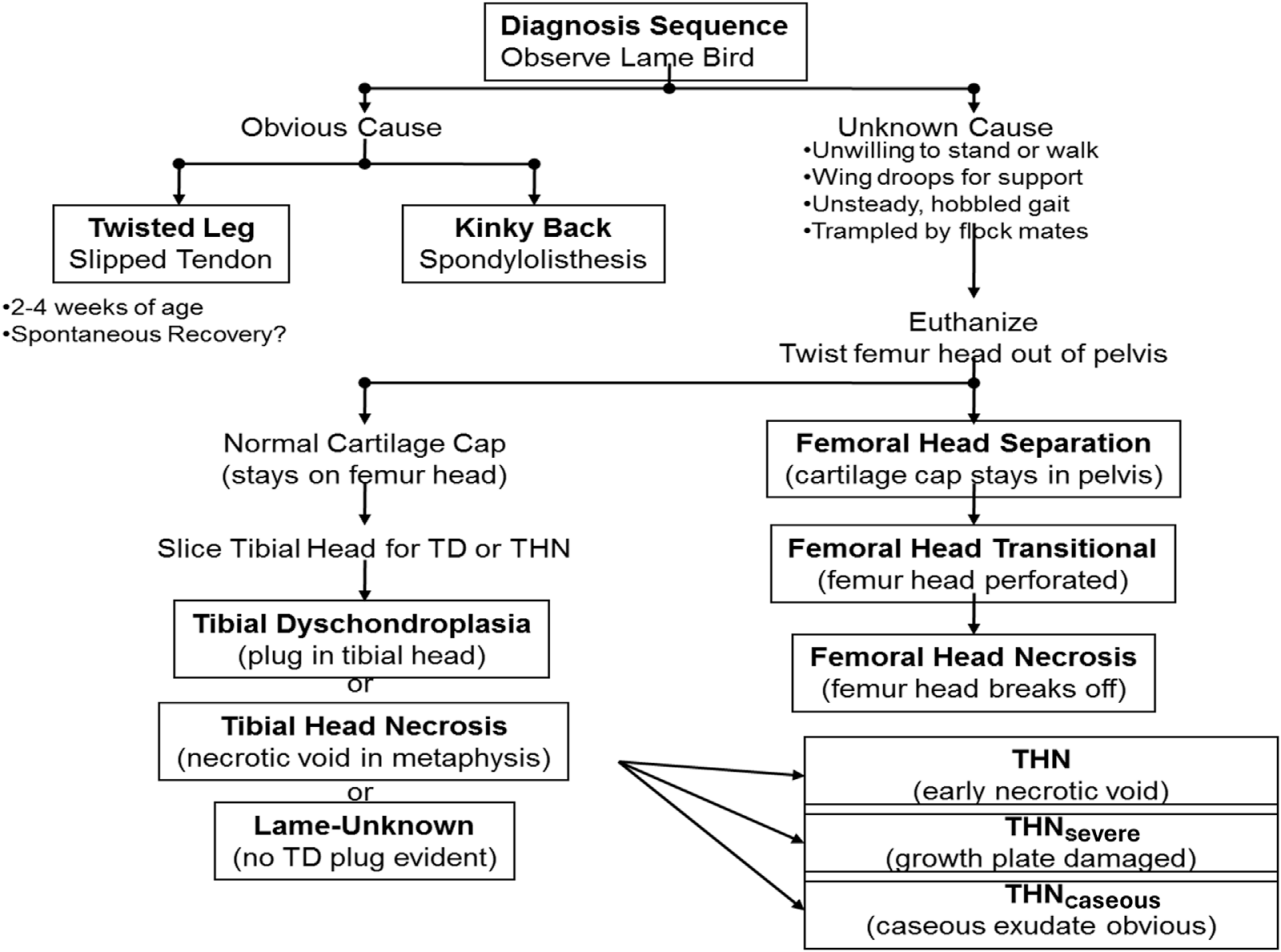
Current process used for BCO diagnosis. Once a lame bird is observed, clinical symptoms are addressed. From this information, it is determined whether the bird has spondylitis/spondylolisthesis, a twisted leg, or must be euthanized and necropsied to gather further information.

Clinical diagnosis of abnormal behavior, morbidity, and lameness is essential as the first step to address BCO-associated lameness as pathogenesis and pathophysiology of the disease take place almost entirely internally within the animal. Clinical symptoms of BCO-associated lameness often include awkward gait, limping, wing dropping to support the body, hesitancy to move, or even complete inability to walk when gently prompted (Wideman, 2016; Al-Rubaye et al., 2017; Alrubaye et al., 2020; Alharbi et al., 2024a; Asnayanti et al., 2024b; Asnayanti et al., 2024c; Do et al., 2024). In rarer cases of severe spondylitis (also known as “kinky back”) the bird will typically be sitting with its legs extended from paralysis, unable to walk and correct to an upright posture (Figure 2). Birds experiencing symptoms of lameness often have great difficulty reaching feed and water sources, which leads to dehydration, impaired growth, and eventual death. Clinical diagnosis of lameness must be addressed as early as possible to maintain the highest standard of animal health and welfare, as well as accuracy of BCO assessment. Typically, BCO diagnosis is conducted via *postmortem* necropsy to assess femoral and tibial lesions, as outlined in Figure 3.

### 3.1 Femoral lesions

**FIGURE 4:**
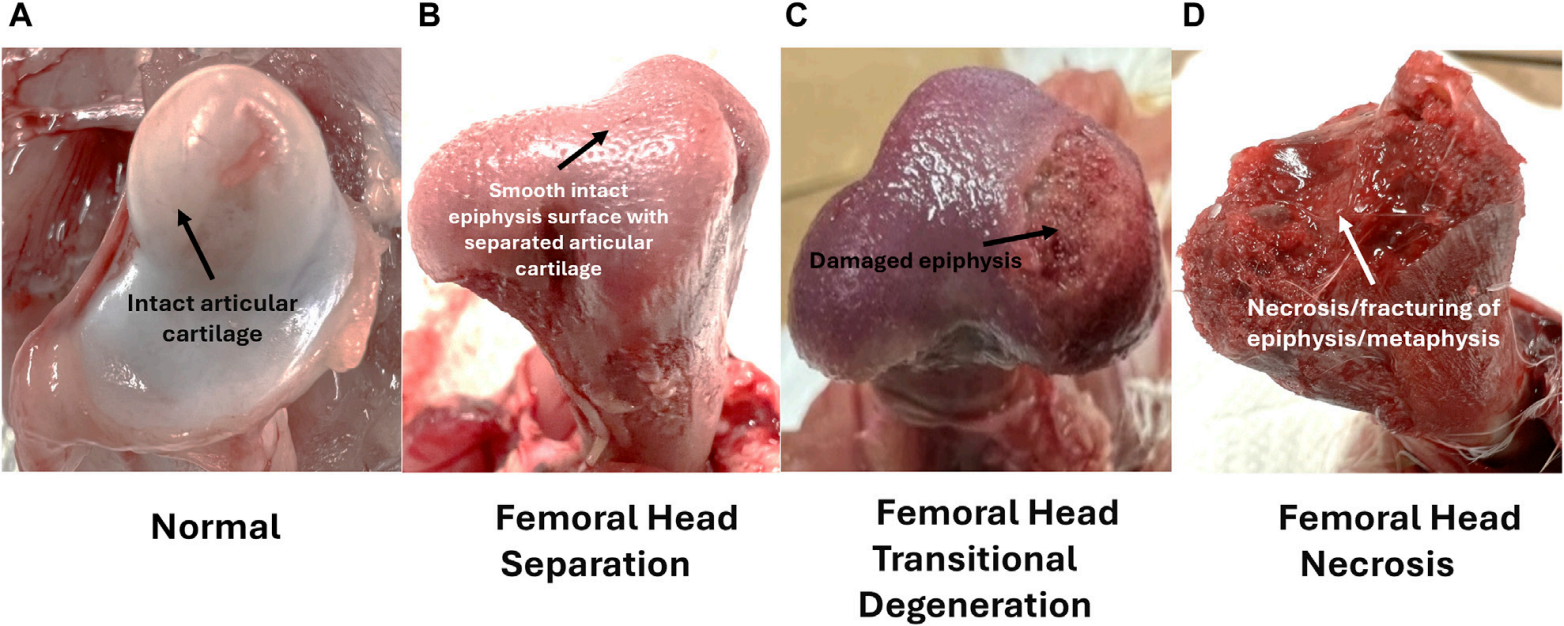
Left to Right; **(A–D)** Femoral ratings for birds diagnosed as lame. Femoral lesion categories are ordered with increasing severity, with arrows marking diagnostic characteristics–**(A)** Normal femoral head with intact cartilage; **(B)** Femoral Head Separation with disarticulated cartilage and smooth intact epiphyseal surface; **(C)** Femoral Head Transitional Degeneration with disarticulated cartilage and somewhat damaged epiphyseal surface; **(D)** Femoral Head Necrosis with extensive damage to the epiphysis and fracturing of the physis. Adapted from Do et al. (2024).

Disease progression of femoral head lesions commonly associated with BCO lameness is shown in Figure 4.

A combination of compromised blood flow, bacterial infection, and mechanical stress leads to lesions in the femoral head, which is diagnosed by *postmortem* disarticulation of the proximal head from the acetabulum for assessment (Wideman, 2016). Lesion categories for the proximal femur head, from least severe to most severe include normal (N), femoral head separation (FHS), femoral head transitional degeneration (FHT), and femoral head necrosis (FHN) (Dinev, 2009; Wideman, 2016; Do et al., 2024). As shown in Figure 4, worsening disease progression states are indicated by several hallmark characteristics as the healthy proximal femoral head transitions from a healthy state, complete with intact articular epiphyseal cartilage and smooth joint articulation, to the FHN stage. Starting with FHS, mechanical stress exacerbated by the bird’s heavy weight leads to the erosion or disarticulation of the epiphyseal cartilage, which usually remains in the acetabulum, exposing the epiphysis to further damage as the bird locomotes. In the transitional stage of FHT, the effects become even more evident, with the epiphysis exhibiting a significant amount of damage. Finally, once disease progression reaches the terminal FHN stage, its necrosis and complete fracturing of the femoral head upon disarticulation renders the bird almost completely unable to move. While transitional states (FHS and FHT) are arguably less extensive in damage to the femoral epiphysis compared to FHN, afflicted birds all exhibit great difficulty in mobility that can be clinically diagnosed with an awkward gait, reluctance to walk, or even tonic immobility (Wideman, 2016; Alrubaye et al., 2020; Alharbi et al., 2024a; Asnayanti et al., 2024b; Do et al., 2024). Most importantly, damage to the exposed epiphysis in all disease progression stages allows for translocated bacterial infection to take place as discussed, leading to increased chances of worsening BCO-associated pathological lameness (McNamee et al., 2000; Wideman, 2016; Alrubaye et al., 2020; Choppa and Kim, 2023).

### 3.2 Tibial lesions

**FIGURE 5:**
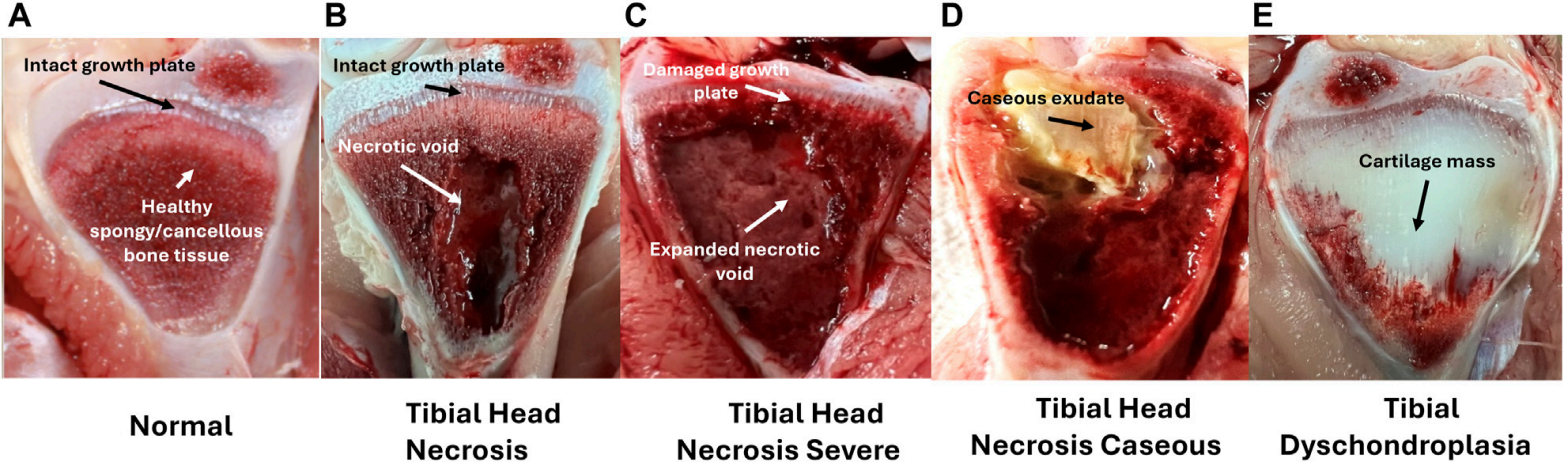
Left to Right; **(A–E)** Tibial ratings for birds diagnosed as lame. Tibial lesion categories are ordered with increasing severity (excluding E) with arrows marking diagnostic characteristics–**(A)** Normal tibial head with intact growth plate and firm, healthy cancellous bone tissue (2); **(B)** Tibial Head Necrosis with intact growth plate and developing necrotic void in cancellous bone tissue; **(C)** Tibial Head Necrosis Severe with damaged growth plate and large necrotic void in cancellous bone tissue; **(D)** Tibial Head Necrosis Caseous with caseous exudate, possibly marking bacterial infiltration site; **(E)** Tibial Dyschondroplasia with accumulated non-vascularized, non-mineralized cartilaginous mass replacing cancellous bone tissue. Adapted from Do et al. (2024).

Much like femoral lesions, tibial lesions result from the combination of mechanical stress and bacterial infection. However, most of the damage is observed by dissecting the proximal tibial head to expose a cross-section of the growth plate. Tibial lesion progression commonly associated with BCO lameness is depicted in Figure 5.

**FIGURE 6:**
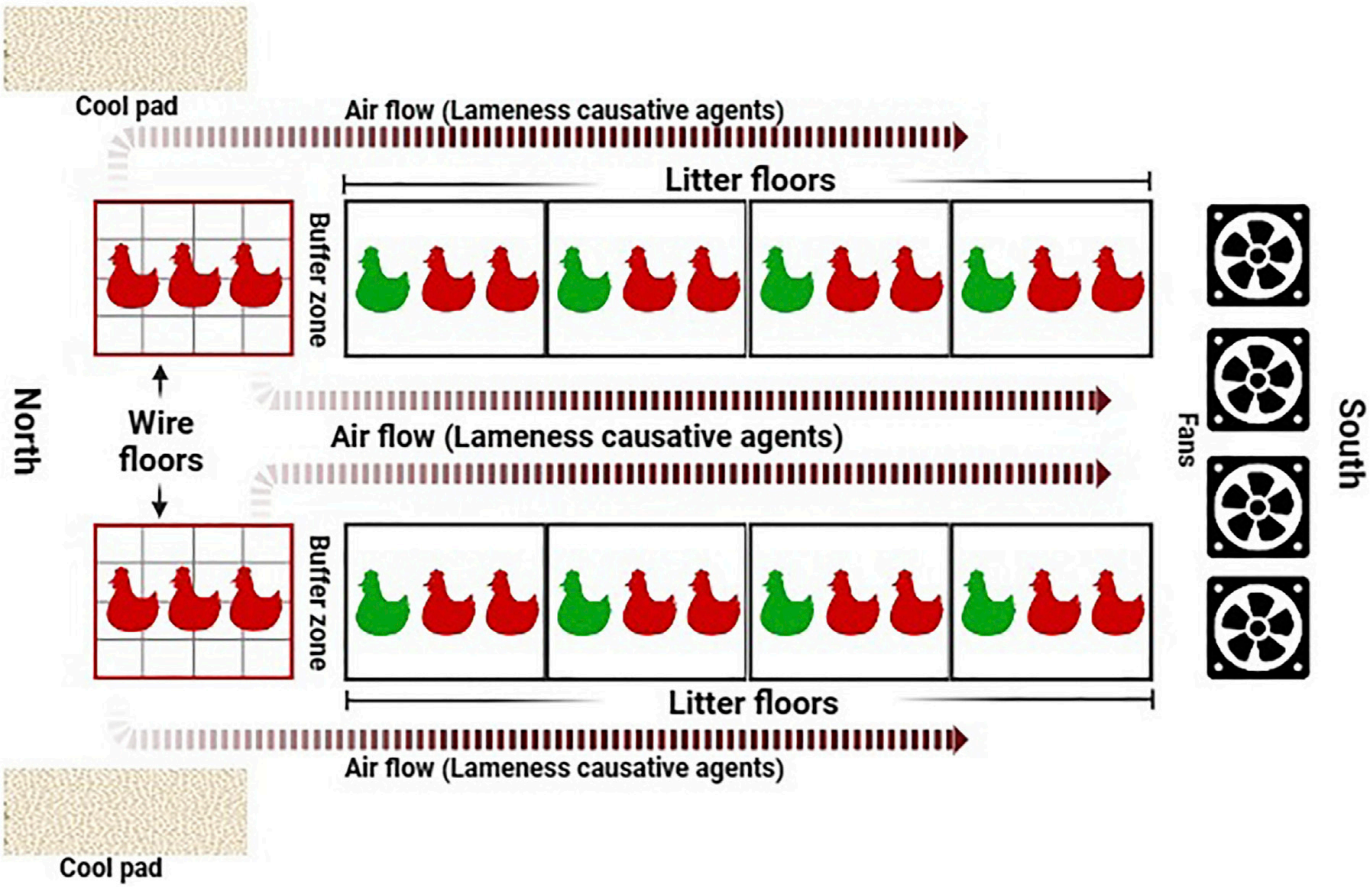
Schematic of the aerosol transmission model. Airflow facilitated by automated mechanical ventilation carries upwind aerosolized BCO etiological agents, released by stressed birds reared on wire flooring, and spreads them to remaining litter-floored birds in the same housing environment. Reproduced from Asnayanti et al. (2024a) with permission from Elsevier.

As characterized by Wideman Jr et al. (2012) and similarly by Do et al. (2024), tibial lesions are also categorized based on severity, ranging from normal (N), tibial head necrosis (THN) to tibial head necrosis severe (THNS). In rare cases, lesions presenting with caseous exudate are also categorized as tibial head necrosis caseous (THNC) (similar to tibial head necrosis sever with a large necrotic void and growth plate damage, but additional caseous bacterial infection appearing as a bright yellow section within the tibial head). As shown in Figure 6, normal healthy tibial cross-sections are presented with an intact and clearly defined metaphyseal growth plate along with firm cancellous (spongy) bone. With disease progression taking place, necrotic degeneration of the cancellous bone leads to soft lesions and necrotic voids (THN) that may increase in size and encroach upon the growth plate, often with visible clefts (THNS) at its most severe stage. In the case of THNC, a cheese-like caseous exudate can often be observed in close vicinity of necrotic lesions, which may mark possible sites of bacterial deposit and infection. Occasionally, tibial cross-sections may also present overgrowths of cartilaginous tissue replacing cancellous bone growth in birds afflicted with tibial dyschondroplasia (TD), caused by chondrocyte death due to insufficient blood supply to the region (Huang et al., 2019). While not directly linked to BCO pathophysiology, TD is also a significant cause for concern in the poultry industry as it can also lead to severe broiler lameness and reduced welfare (Sanotra et al., 2001; Bradshaw et al., 2002).

### 3.3 Vertebral lesions (spondylitis)

In addition to lesions typically found in the proximal femoral and tibial heads, broilers suffering from BCO can also develop vertebral lesions in the same manner as that seen in leg bones–via hematogenous distribution of translocated pathogenic bacteria into osteochondrotic clefts in the damaged thoracic vertebrae from mechanical stress, causing swelling and abscessation of the fourth vertebral body (T4) (Wideman, 2016). Broilers affected by BCO vertebral lesions (or spondylitis) can be clinically diagnosed by the hallmark hock recumbency posture and paralysis, causing a complete inability to maintain an upright posture in these animals. While the presenting clinical symptoms of BCO-associated vertebral lesions are almost identical to that of spondylolisthesis, which is often referred to as “kinky back”, the latter is usually noninfectious in nature compared to infectious spondylitis, and care must be taken in diagnostic evaluation of this specific clinical symptom with regards to vertebral BCO/spondylitis (Duff, 1990; Dinev, 2012; Robbins et al., 2012; Dinev, 2014; Borst et al., 2017). Similar to leg bone lesions caused by the disease, spondylitis causes severely reduced animal welfare standards from the inability to access feed and water, and results in culling of afflicted birds. In addition to species commonly associated with femoral and tibial lesions such as *Staphylococcus* spp., there is a wide breadth of reports in current literature attributed to Enterococci in poultry spondylitis, with pathogenic *Enterococcus cecorum* as a major etiological agent throughout the years (Stalker et al., 2010b; Robbins et al., 2012; Dunnam et al., 2023). Origination, lineage, and pathogenicity of pathogenic strains of interest–which differ significantly to their commensal counterpart (Borst et al., 2015) – remain under investigation, with a previous work from Laurentie et al. (2023) suggesting a common lineage among poultry pathogenic isolates. Findings from Borst et al. (2017) also highlighted a significant impact of early *E. cecorum* infection, coupled with a natural susceptibility to osteochondrosis dissecans lesions in the free thoracic vertebrae of the broiler, in spondylitis development. More recently, Rhoads et al. (2024) identified a possible polyphyletic origin to pathogenic isolates associated with BCO lameness and reported outbreaks in recent years using publicly available *E. cecorum* genomes, with pathogenicity suggested to have been caused by mutations rather than gene acquisition, though–as the authors have noted–much more evidence collection and analyses remain to determine pathogen genomic diversity in reported outbreaks within the same facility, as well as a pressing need for expansion of genome databases, which are currently poorly documented.

## 4 Findings and recent advancements

To this day, Wideman (2016) and Wideman and Prisby (2013) still constitute some of the most exhaustive and documented sources in BCO pathophysiology and clinical diagnosis of the disease, both from past literature and the authors’ own bodies of work. Additionally, Wideman et al. (2012) also introduced the first highly effective wire-flooring model to induce BCO lameness in experimental broilers, which had traditionally been accomplished–albeit to varying degrees of success–via injection of *Staphylococcus* spp. to induce bacteremia and subsequent osteomyelitis (Emslie and Nade, 1983; Daum et al., 1990). This model leverages the postulated BCO pathophysiology, which involves the stress imposed on immature leg bones by the animal’s weight and exacerbated by the uneven wire flooring the animals traverse on without direct handling by research personnel, thus eliminating the stress from this factor (Wideman Jr et al., 2012). Whether in its original iteration (Ramser et al., 2021; Weimer et al., 2021) or as an incorporation into other approaches (Asnayanti et al., 2024b; Greene et al., 2024), the wire flooring remains a gold standard in reliable induction of experimental BCO-associated lameness. These foundational findings have equipped researchers with valuable insights, upon which constant findings and advancements pertaining to various aspects of BCO-associated lameness have been made, and will continue to be explored in this relatively young field of research.

### 4.1 A novel BCO induction model

In the years following findings from Wideman et al. (2012), investigation of novel approaches to BCO induction model and other mitigatory measures–mainly in the form of feed additives and supplements–have taken place. Expanding upon the effective wire-flooring model, Asnayanti et al. (2024b) developed the aerosol transmission model, the schematic of which is shown in Figure 6:

While the wire-flooring model is highly effective, being able to induce over 70% of cumulative lameness incidence in the respective animals (Wideman et al., 2012; Wideman, 2016; Al-Rubaye et al., 2017) it is also aggressive and unrepresentative of normal rearing conditions, thus potentially reducing research translatability into a practical industrial setting. Moreover, fabrication and breakdown of wire-flooring pens is a highly laborious and time-consuming process, which makes accessible experimental replication a challenging task. The novel approach of the aerosol transmission model seeks to improve these aspects. Relying on aerobic horizontal transmission of aerosolized etiological agents from stress-imposed, wire flooring-reared seeder birds to others within the same housing environment, this induction model resembles infectious transmission and outbreaks commonly seen within poultry houses with active ventilation (Adell et al., 2015; Elliott et al., 2019). As validated through a series of trials, the aerosol transmission model has been shown to be capable of inducing experimental lameness incidence rates comparable to the wire-flooring model, which range from 60% to 73% of experimental animals (Asnayanti et al., 2024b), thus providing a reliable alternative induction method that further expands the repertoire of BCO research methods alongside the wire-flooring model and experimental inoculation.

### 4.2 Mitigatory strategies

As briefly discussed, the productive life cycle of conventional meat broilers is incredibly short compared to other livestock species, averaging 6 weeks of age at processing. An inability to effectively treat individual animals in the field due to flock turnaround on such a short time frame has led to immediate culling of lame birds–BCO-associated or otherwise–as the only appropriate response to negative animal welfare and production efficiency (Mitchell, 2014; Granquist et al., 2019). From an integrator’s perspective, early preventive and mitigatory strategies, such as feed additives and supplements, along with good management practices have become increasingly important to protect flocks from significant economic losses due to BCO lameness. It has been reported that supplementation of high-quality essential trace minerals such as copper, manganese, and magnesium in the broiler diet may have a positive effect in the strengthening and increased expression of TJ proteins, leading to decreased chances of translocation events and subsequent clinical BCO lameness incidence (Alrubaye et al., 2020). Other prophylactic dietary supplements, such as 1, 25-dihydroxyvitamin D_3_-glycosides and Fe-CNP (iron-loaded chitooligosaccharide nanoparticles) have also demonstrated positive outcomes in clinical BCO lameness incidence reduction (Yousefi and Saki, 2019; Asnayanti et al., 2024a). Similarly, a recent study using isolated environmental chambers by Do et al. (2024), which evaluated prophylactic spraying of day-of-hatch chicks with probiotic *Enterococcus faecium* on the same lameness reduction parameters has revealed positive results, which also suggests a dose-dependent effect of the examined probiotic. Interestingly, supplement inclusion timing has consistently revealed a trend across different types in the overall outcome regarding lameness reduction rate, with early supplementation–usually the first 4 to 6 weeks of the experiment–often conferring the same performance as that throughout the entire experimental period, compared to inclusion within the latter half (Alharbi et al., 2024a; Asnayanti et al., 2024a). While the modes of action in these supplements are continuously under investigation, these findings are consistent with the well-established developmental timeline of physiological systems across species, including gastrointestinal and skeletal, where early influence of various nature on the respective immature environments often has lasting impacts as the organism grows–positively or negatively (Montgomery et al., 1999; Karsenty and Wagner, 2002; Di Mauro et al., 2013; Van Oudenhove et al., 2016).

### 4.3 Molecular investigation

In terms of molecular underpinnings, evidence of various cellular functional dysregulation has been implicated in the pathogenicity of BCO lameness. For example, signs of mitochondrial dysfunction, including increased mitochondrial biogenesis responses (maintained by PGC-1α and PGC-1β), increased trend of mitochondrial fission/fragmentation state (OMA1) rather than fusion state (OPA1), and decreased mitochondrial respiratory activity (regulated by FOXO3, FOXO4, and av-UCP) as well as functional genes (ANT, COXIV, and COX5A), have been documented in BCO-affected tissue (Ferver et al., 2021). Results from Ramser et al. (2022) also suggest dysregulation in autophagic mechanisms and downregulation of genes associated with autophagy (ATG13, SQSTM1, ATG9B, ATG16L, ATG12, LC3C and RAB7A) – which are crucial in maintaining cellular homeostasis, elimination of foreign entities, and have been previously linked to various skeletal diseases and disorders (Liu et al., 2014; Ichimiya et al., 2020) – leading to decreased cell viability after *in vitro* inoculation of the known BCO isolate *S. agnetis* 908, in a similar fashion to direct autophagy inhibition via exposure to 3-Methyladenine and chloroquine. These results also offer an insight into possible mechanisms through which bacterial infection influences and exacerbates colonization sites that eventually lead to increasing severity and lameness, an aspect that has not yet been clearly defined in literature. Finally, high-throughput metabolomics has recently been employed in examination of dysregulated metabolic cascades and their outcomes in BCO-affected tissue (Ramser et al., 2023) with metabolite profile from affected bones showing distinctive differences compared to that seen in healthy bone tissue, including activation of CD40 and AQP7 – the former of which has been associated with decreased bone mineral density and *S. aureus* bacterial infection, both major contributors to BCO etiology (Pineda et al., 2011; Schlievert et al., 2019). While much further research is needed for a complete picture regarding BCO pathogenesis and pathophysiological changes on the molecular level, these early encouraging results will nonetheless be invaluable in future research direction.

## 5 Industrial concerns of BCO lameness

As mentioned, due to its increasingly overwhelming and widespread impact, the issue of bacterial chondronecrosis with osteomyelitis is accompanied by a wide range of concerns within the poultry industry. Alongside major animal welfare issues and tremendous economic losses from impacted birds due to mortality and carcass condemnations, BCO and its associated lameness may also be implicated in other aspects of poultry meat production–such as meat quality and food safety.

### 5.1 Economical concerns

Lameness is often regarded as the main source of economic loss in meat birds as it not only results in bird loss, but can negatively impact the growth and performance of meat birds, leading to reduced productivity and economic losses (Pearce et al., 2023). In 2022, the USDA reported 9.17 billion broilers produced in the United States, and BCO can be linked to a loss of between $80 million and $120 million in sales (Granquist et al., 2019; Adnan Yousaf et al., 2021). Modern industrial broiler production often relies on an “all in, all out” model, in which a broiler flock of a similar age/background goes through production stages at the same time with housekeeping downtime between flocks (Biosecurity Nova Scotia, 2020; Brothers, 2022). This model allows greater control from the integrator in maintaining biosecurity and reducing transmission of diseases among animals due to a lack of mid-flock movement and the possibility of thorough cleanout between flocks, which is extremely important in cases of disease outbreaks that may require thorough containment and disinfection–or depopulation of the entire flock as mandated by regulatory bodies (UC Davis Veterinary Medicine, 2019; APHIS, 2022). Despite the advantages offered, however, an inherent downside to this model lies in the turnaround speed at which a flock enters and leaves a complex that does not allow for isolation of affected individuals and prolonged disease treatment plans like other major livestock species while maintaining productive efficiency (LeBlanc et al., 2006; Seaman and Fangman, 2023). Considering the intractably internal nature of BCO pathogenesis and pathophysiological changes, as well as this lack of individual treatment plans for birds with an already incredibly short productive life cycle, there has yet been an effective therapeutic treatment for BCO as of current, placing the onus on preventive measures and strategies instead–or prompt culling of birds exhibiting clinical signs, resulting in animal losses. While several preventive approaches using dietary supplements (Alrubaye et al., 2020; Alharbi et al., 2024a; Asnayanti et al., 2024c) and early spraying of a probiotic strain (Do et al., 2024) have shown promising results in reduction of observable clinical BCO-associated lameness diagnosis, much more work remains to be done to find a potential cure. In addition to the loss in meat production, manually assessing individual poultry in a commercial setting is infeasibly time-consuming and labor-intensive, as a standard grow out barn is commonly responsible for approximately 36,000 to 52,000 broiler heads produced every 42 days on average, a complex of which may consist of multiple houses on the same productive timeline (Brothers, 2022). Moreover, extensive worker handling could cause undue distress to birds (Pearce et al., 2023), leading to further productivity and time loss, as well as potentially increased broiler injuries and lameness.

### 5.2 Animal welfare concerns

Discussions of poultry welfare would not be complete without extensive consideration of leg issues and lameness as an overarching topic. In addition to several common leg or foot conditions caused by inadequate housing and nutritional management such as foot pad dermatitis and hock burns (Berg, 2004; Shepherd and Fairchild, 2010), weight-induced lameness–whether through developmental issues, mechanical injuries, or bacterial infection as a cause–contributes to a significant part of this conversation. Broilers afflicted with increasing degrees of lameness have been observed to spend much less time walking, standing upright, with fewer feeding visits compared to healthy birds (Weeks et al., 2000; Nääs et al., 2009) which are all associated with reduced productivity and welfare standards. Similarly, not only is BCO-associated lameness considered painful to the animal and predisposes them to the same lameness-related issues, but the condition also leads to loss of marketable products from culls and condemnations (Granquist et al., 2019). Furthermore, as a species with an established hierarchy, broilers outwardly exhibiting clinical lameness will frequently experience aggression from more dominant littermates, pushing them further down the social “pecking order” within the flock and exacerbating negative welfare impacts (Favati et al., 2014a; Favati et al., 2014b; Marino, 2017). As another unfortunate consequence of this innate behavior, some broilers may modify their behavior to “mask” any apparent indication of pain and avoid flock hostility, which greatly contributes to the difficulty in accurate diagnosis of BCO-associated lameness–or other lameness evaluations in general–outside of clinical diagnosis as suggested by several studies indicating BCO lesion development even in apparently healthy birds (Alharbi et al., 2024a; Asnayanti et al., 2024b). Today, lameness is one of the most important welfare concerns for the poultry industry, particularly with regards to broilers (Granquist et al., 2019). Alongside the obvious negative impacts to animal health, consumers are increasingly becoming aware of industrial practices and animal welfare impacts in agricultural production (Estes and Edgar, 2013). As such, addressing lameness remains a top priority for the poultry industry to improve consumer perception and confidence, an aspect it has constantly striven to achieve (McCulloch, 2017; Sinclair et al., 2019; Sonntag et al., 2019). Kapell et al. (2012) and Siegel et al. (2019) highlighted genetic improvements to skeletal strength and integrity made within the past 2 decades, which have resulted in encouraging findings regarding decreased heritability and prevalence of common leg disorders such as long bone deformation (valgus/varus, bowed legs), and tibial dyschondroplasia while maintaining a similar level of performance increase. Despite this, current literature remains quite lacking in studies examining whether such genetic improvements have resulted in any appreciable effects on bone parameters associated with BCO pathogenesis and pathophysiology. Interestingly, a study by Chen et al. (2018) has found that osteochondrotic severity remains similar between a random-bred line representative of 1950s broilers and several modern broiler lines–with the former weighing significantly less during development–suggesting such a condition may not be weight-dependent. It is clear that much more research in this aspect is warranted for a thorough understanding of BCO pathology and effective breeding selection strategies, as well as management practices to decrease its prevalence.

### 5.3 Possible links to meat safety and quality concerns

The quality and safety of poultry products remain a major concern for both consumers and producers (Rouger et al., 2017). In terms of microbial contamination and food safety, risks of salmonellosis have become an assumed facet of poultry meat and eggs (Morris and Wells, 1970; Whiley and Ross, 2015; Antunes et al., 2016) that necessitates thorough rules and regulations regarding poultry meat and egg handling because of the massive and highly complex scale of poultry production and processing in the United States. Just as frequently, contamination of enterococci and staphylococci from the animal’s gut has also been reported due to lower processing standards compared to other livestock species (Bortolaia et al., 2016). Considering the etiology, pathogenesis, and pathophysiology of BCO, there are grounds for heightened attention regarding poultry meat contamination from potential internal lesions that have gone undetected during carcass processing, especially in birds that do not show the associated clinical symptoms essential to pre-mortem BCO-associated lameness diagnosis (Alharbi et al., 2024a; Asnayanti et al., 2024b).

**FIGURE 7:**
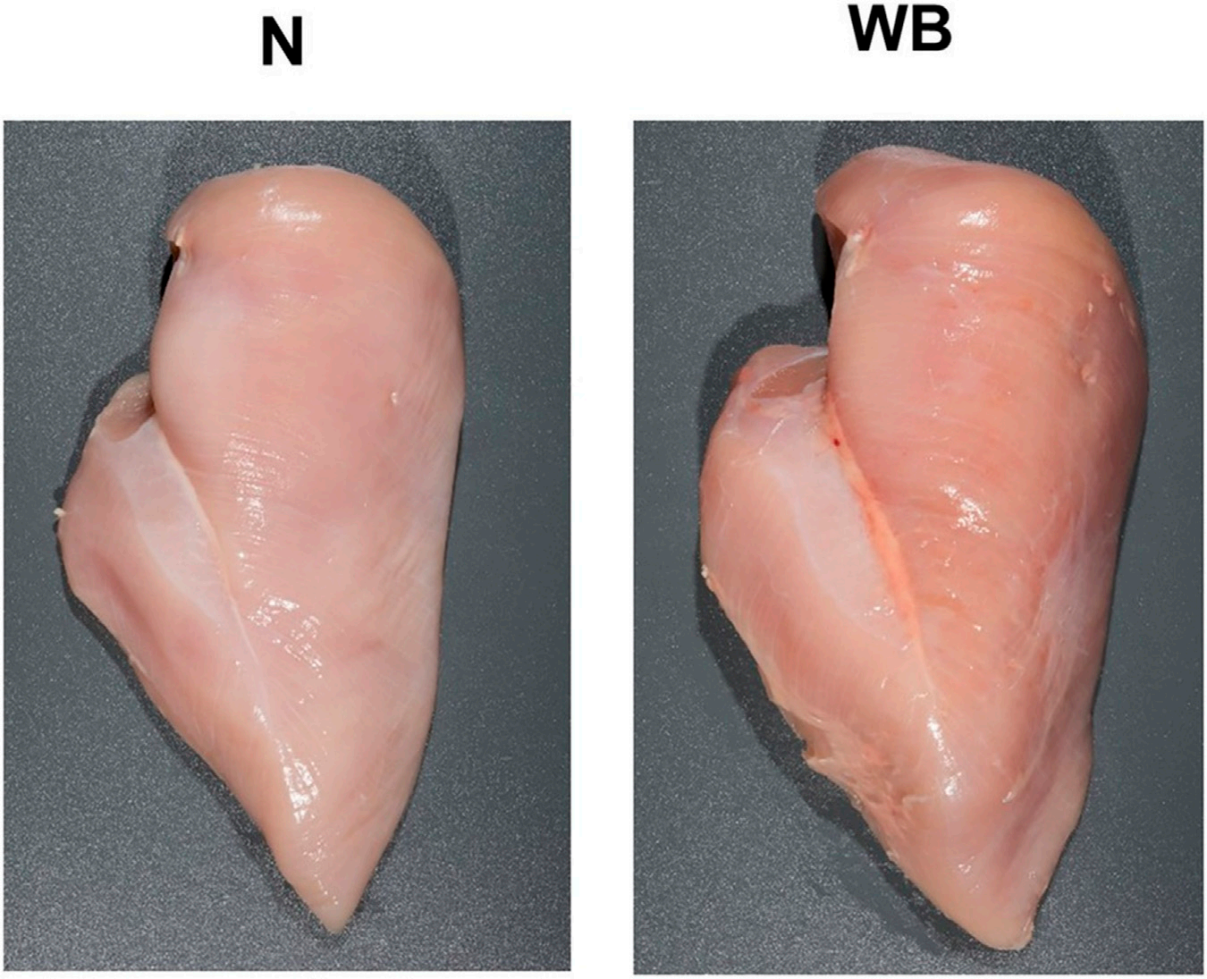
Exhibition of a normal (N) broiler pectoralis major muscle compared to one afflicted with woody breast syndrome (WB). Note the visually enlarged, darker, and firmer texture of the cranial region and caudal pronounced caudal ridge region of WB in comparison to N. Reproduced from Welter et al. (2022).

**FIGURE 8:**
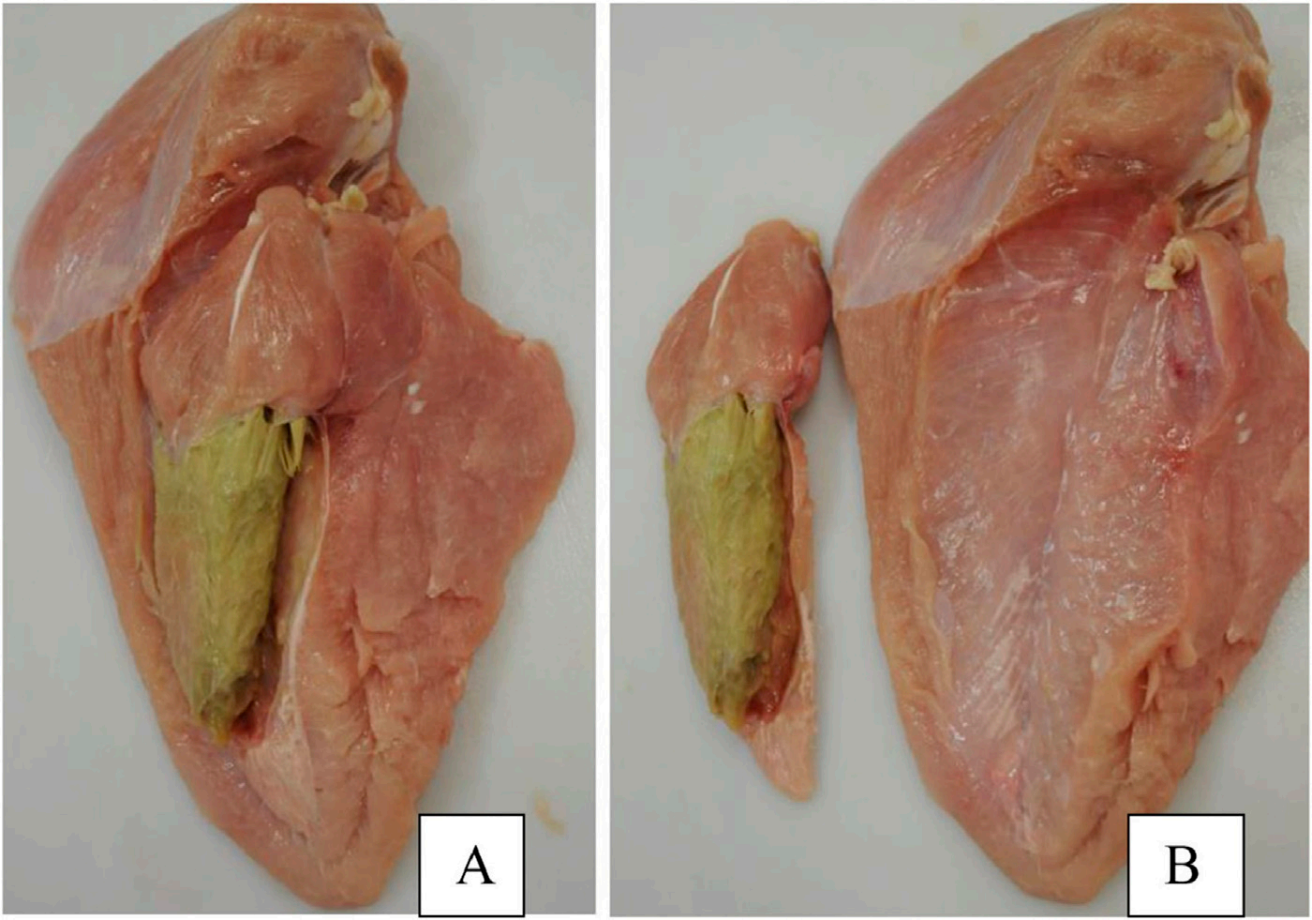
Exhibition of the pectoralis minor afflicted with severe deep pectoral myopathy (DPM). **(A)** Breast complex including both the pectoralis major and pectoralis minor muscles. **(B)** Separation of the complex. Reproduced from Giampietro-Ganeco et al. (2022) with permission from Elsevier.

Product quality from injured or affected animals is often influenced by excess inflammation in the tissue around wound sites (Gifford et al., 2012). Due to the damage of connective tissue surrounding affected joints, the integrity of the meat structure may also be compromised, which is not only a loss of product, but is often unappealing to consumers (Lee and Mienaltowski, 2023). While it is unclear whether BCO and its associated lameness may have a significant direct impact on issues pertaining to meat quality aside from these points of consideration, such a dialogue may still benefit from a broader point of view with regard to the physiology of the modern broiler. Having become a “hot button” topic of discussion since their rising prevalence in the past decade, pectoralis major muscle myopathy (woody breast–WB, Figure 7) and deep pectoral myopathy (green muscle disease–DPM, Figure 8) are some of the most recognizable examples of degenerative myopathies miring product quality within the industry that have been linked to the heavy weight and rapid muscling of commercial broilers (Bilgili and Hess, 2008; Sihvo et al., 2018).

Breast muscle affected by WB syndrome is characterized by an abnormally tough and rubbery texture, usually with high calcium content (Welter et al., 2022), resulting in poor palatability for the consumer. Several postulated pathogenesis and pathophysiological changes have been proposed in current literature of WB syndrome. Hoving-Bolink et al. (2000) entertained the possibility of switching toward glycolytic pathways in muscle development of the animal to fit selection pressure for higher breast yield and efficiency, which could potentially lead to higher development rates of muscular abnormalities as suggested by Petracci and Berri (2017). At the same time, decreased blood and oxygen supplies to muscle tissue due to poor vasculature may also contribute to the issue, as evident by a decrease in myopathy severity as muscle tissue is examined moving inward relative to the pectoralis major’s surface (Hoving-Bolink et al., 2000; Sihvo et al., 2018). Finally, several studies have also explored and suggested the possibility of metabolic waste product displacement playing a significant role, which introduces highly oxidative and stressful internal conditions for cellular functions, resulting in inflammation, mitochondrial damage and dysfunction, and prompting regenerative processes that ultimately lead to characteristics distinctive to growth abnormalities associated with woody breast (Boerboom et al., 2018; Papah et al., 2018; Sihvo et al., 2018). While no singular definitive cause has been determined, it is accepted that WB is a multi-faceted issue with wide reaches to fast-growth genetics, nutrition, and growth management of heavier birds (Caldas-Cueva and Owens, 2020). On the other hand, DPM is characterized by a hidden unappealing “greening” (necrosis) of the deep pectoralis minor muscle (tender) that would escape detection in unfabricated broiler carcasses. The disease is caused by excessive wing-flapping in heavier birds, leading to blood supply cutoffs and injuries to the pectoralis minor muscle as the superficial pectoralis major grows in size and resulting in necrosis of the former (Bilgili and Hess, 2008). Despite these inherent differences in pathogenesis between discussed myopathies and BCO and their impact on broiler meat quality, the heavy body weight and rapid muscling of the modern broiler seem to be the singular converging factor that leads to physiological strain on both breast muscle and leg bones, resulting in their respective associated pathologies. Indeed, reports of all three conditions are much scarcer in layers, which are much smaller and slower in growth rates compared to their meat broiler counterparts. In this sense, a movement toward reduced broiler sizes, achievable through limitations on the maximum potential genetic growth such as those implemented in European markets (Bos et al., 2018; Tixier-Boichard, 2020) could be the answer to improving meat quality and reducing BCO incidence at the same time. Of course, careful considerations must be taken with this approach to maintain production efficiency, profits, and product affordability for the consumers, as well as the potential environmental impacts of such changes (Leinonen et al., 2012). Evidently, it would be remiss to acknowledge such a connection between these conditions at face value due to a severe gap in current literature regarding this topic, where incidence of BCO-associated lameness and common breast myopathies are concurrently examined. Inherent factors contributing to such a dearth of knowledge include the different models employed in the respective line of research, as well as the expertise necessary for rapid accurate diagnosis and evaluation of the associated pathologies (Pang et al., 2020; Hisasaga Jr, 2021). However, science is a collective of collaborative efforts, and therein lies a significant opportunity for researchers in both fields to contribute to the overall understanding of these respective pathologies and possible interactive effects between them.

## 6 Conclusion

Bacterial chondronecrosis with osteomyelitis poses a formidable challenge to avian health and welfare, with potentially dire implications for poultry meat safety and quality, necessitating comprehensive strategies for prevention, diagnosis, and management. Collaboration between all aspects of the industry, including primary breeders, commercial producers, veterinarians, and research bodies is essential to addressing the ever-present issue of lameness. By understanding the complexity of this condition, its pathology, and learning to assess affected birds, producers can better manage flock health and condition as well as optimize production through pre-emptive preventive measures. Most importantly, in prioritizing the animal’s health and welfare by keeping an open-minded point of view, poultry producers will be better equipped for dialogues regarding the future direction of the modern broiler, greatly aiding the sustainability and resilience of the industry in its continuous goal of providing the growing populace with an affordable, safe, and high-quality source of animal protein.
